# Trends in American scientists’ political donations and implications for trust in science

**DOI:** 10.1057/s41599-022-01382-3

**Published:** 2022-10-13

**Authors:** Alexander A. Kaurov, Viktoria Cologna, Charlie Tyson, Naomi Oreskes

**Affiliations:** 1 Department of the History of Science, Harvard University, Cambridge, MA USA; 2 Program in Interdisciplinary Studies, Institute for Advanced Study, Princeton, NJ USA; 3 Blue Marble Space Institute of Science, Seattle, WA USA; 4 Department of English, Harvard University, Cambridge, MA USA

**Keywords:** Politics and international relations, Science, technology and society, History

## Abstract

Scientists in the United States are more politically liberal than the general population. This fact has fed charges of political bias. To learn more about scientists’ political behavior, we analyze publicly available Federal Election Commission data. We find that scientists who donate to federal candidates and parties are far more likely to support Democrats than Republicans, with less than 10 percent of donations going to Republicans in recent years. The same pattern holds true for employees of the academic sector generally, and for scientists employed in the energy sector. This was not always the case: Before 2000, political contributions were more evenly divided between Democrats and Republicans. We argue that these observed changes are more readily explained by changes in Republican Party attitudes toward science than by changes in American scientists. We reason that greater public involvement by centrist and conservative scientists could help increase trust in science among Republicans.

## Introduction

American academia is often accused of liberal bias, and some observers have blamed the academy’s left-wing slant for undermining trust in science (Duarte et al., 2015). The impression that scientists are overwhelmingly liberal has provided an impetus for conservative attacks on scientific findings and fueled the rise of conservative counter-institutions dedicated to challenging “liberally biased” academic science, chiefly for a lay audience (Mann and Schleifer, 2020).

Previous attempts to study the political ideologies of academics, however, have suffered from severe limitations. Many studies of faculty politics aiming to unmask professorial leftism—undertaken by think tanks such as the American Enterprise Institute, or conservative academic centers such as the Mercatus Center—are clearly biased, focusing disproportionately on elite private universities, or selectively targeting disciplines such as women’s studies, which has an acknowledged anti-sexist political orientation. Others are methodologically problematic, relying on the crude binary metric of voter registration to try to understand the complex question of political viewpoint, or drawing conclusions from very small sample sizes (Tyson & Oreskes, 2020). Methodologically robust studies that include faculty from a wide range of disciplines and institution types, have a large sample size, and ask standardly worded questions find a more centrist professoriate than is alleged in conservative discourse. The most comprehensive and methodologically robust study of faculty politics, done in 2006 by the sociologists Neil Gross and Solon Simmons, found that moderates slightly outnumber liberals (Gross and Simmons, 2014). This finding does not support allegations of widespread extreme liberal orientation—much less bias—in academic life. Nonetheless, there is evidence that most American scientists favor the Democratic Party; a 2009 Pew survey found that 55 percent of scientists identify as Democrats, while just 6 percent say they are Republicans (32 percent identify as independents) (Rosenberg, 2009).

In this paper, we attempt to better understand the political views of American scientists by examining their contributions to political campaigns, using data-mining techniques to examine publicly available information. The Federal Election Commission (FEC) maintains a publicly accessible database of donations made by individuals to federal candidates and parties from 1979 to the present, which can be sorted according to many parameters, including professional employment. The FEC data, covering 43 years, allow us to track longitudinal as well as short-term shifts in political giving by scientists. This data set has the added advantage of including scientists working in both industry and academia.

Recent years have seen a large increase in total federal donations, many of them small-dollar amounts, driven largely by online-donation platforms and social-media campaigns. This increase in political giving provides a new incentive to look at this body of data, which has previously, for example, been used to assess the political ideologies of American lawyers (Bonica et al., 2015). Donations are of course not the same as beliefs—and those who donate are a subset of all scientists, biased in favor of those motivated to make a monetary donation—but donations are one measure of beliefs. We use monetary donations to political parties as a clearer measure of political orientation than party registration, insofar as many Americans, including many scientists (according to Pew data), are independent voters, people may not change their registration even as their views may evolve, and/or there may be a lag between evolving views and changing registration. We use monetary donations as a quantifiable measure, as opposed to evolving political views, which may be difficult or impossible to quantify. Moreover, the data on donations is homogeneous across the United States, while voter-registration lists must be requested from each state, and the information shared in them varies from state to state.

## Methodology

FEC donation records are available from 1979 (FEC.gov, 2022). We weigh donations by amount and consider donations to Republicans, Democrats, and all third parties combined. Donations to campaigns and candidates whose affiliation to the political parties is marked “unknown” in the FEC database were excluded. The proportion of unidentified donations is low, and cannot affect the main results of this study. However, in some cases it may be comparable to donations to third parties. From 2002 on, each FEC donation record lists the donor’s employer and occupation. We filter the FEC data by occupation to calculate the fraction of political donations from academic scientists, industry scientists, and engineers.

To capture political donations from academic scientists, we select donors who list any employer that contains “college” or “university” in its name, and select donors whose occupation contains words “professor,” “faculty,” “scientist” or “lecturer.” In order to select a contrasting group, we also select occupations that contain the word “administrator”. We present donations data from all college and university employees, as well as the general professoriate. To isolate the scientists within the professoriate, we have matched donor names to records in Scopus, a large database of scholarly abstracts and citations. We separately consider Ivy League institutions and institutions that are members of the Council for Christian Colleges and Universities.

To capture donations from industry, we focused on ten energy companies—Exxon Mobil, Chevron, Marathon Petroleum, Phillips 66, Valero Energy, Energy Transfer, World Fuel Services, ConocoPhillips, Exelon Corporation and Plains GP Holdings—which rank highly on the S&P 500 and employ many scientists and engineers. To determine which energy-sector donations come from executives and which from working scientists, we filter based on whether the listed occupation contains “supervisor”, “chairman,” “CEO”, “COO,” “VP,” “executive”, or “president” (to capture executives); or “engineer,” “geologist,” “chemist,” “geophysicist,” “scientist,” “professor,” or “researcher” (to capture scientists). The pipeline of the analysis can be accessed on GitHub code repository (https://github.com/lue/political-donations-by-scientists).

To gain information on how Republicans’ and Democrats’ levels of trust in the scientific community have evolved over time, we use the General Social Survey (Smith, 2015), which has been conducted regularly from 1972, with the most recent survey in 2021. To identify the political affiliation of individuals, we use the answer to the question: “Generally speaking, do you usually think of yourself as a Republican, Democrat, Independent, or what?” We omit from our analysis records of individuals who identify with another party, provide no answer, or identify as Independent. To estimate the level of trust in the scientific community, we use the response to the question: “I am going to name some institutions in this country. As far as the people running these institutions are concerned, would you say you have a great deal of confidence, only some confidence, or hardly any confidence at all in them? … K. Scientific Community.” We use the fraction of people who answered “a great deal” to track Republicans’ and Democrats’ views on the scientific community over time.

We adapt Scopus database API (Elsevier, 2022) to retrieve the subject areas of the professors based on their names. This operation allows us to identify whether donors work in physical, health, social or life sciences as defined in the Scopus database. Often researchers have fractional contributions to various fields; in such cases, we proportionally split their donations among corresponding areas. We do not use geographical information of donors with the affiliations in the Scopus database given that people might not live near their place of work, and many academic workers move between institutions. Some common names appear in the Scopus database many times; and in such cases, we take the top search result. In total, we identify approximately 100,000 professors making 1,000,000 unique donations since 2002. With the use of the Scopus database, we are able to identify 80,000 of these professors. Excluding all search results with multiple entries reduces number of uniquely identified professors to 28,000 but does not qualitatively change our results.

We plot lines from the moment when sufficient FEC data becomes available for the given time period to calculate statistics. Thus, lines do not start immediately when data collection was initiated in 1979, or for when the additional employment data began to be included in FEC records in 2002.

There are certain limitations to our approach. First, names and employment information are self-reported and may therefore be incorrect. Second, donation patterns have changed dramatically in recent years due to online-donation platforms that simplified the donation process. Therefore, donation patterns from recent years may not be strictly comparable to earlier periods. Third, scientists who donate are likely to be more politically active than the general population of scientists. We attempt to mitigate these shortcomings by considering only carefully selected groups of donors and looking strictly at the ratio of donations to Republicans and Democrats.

## Results

### Scientists in Academia

**Fig. 1:**
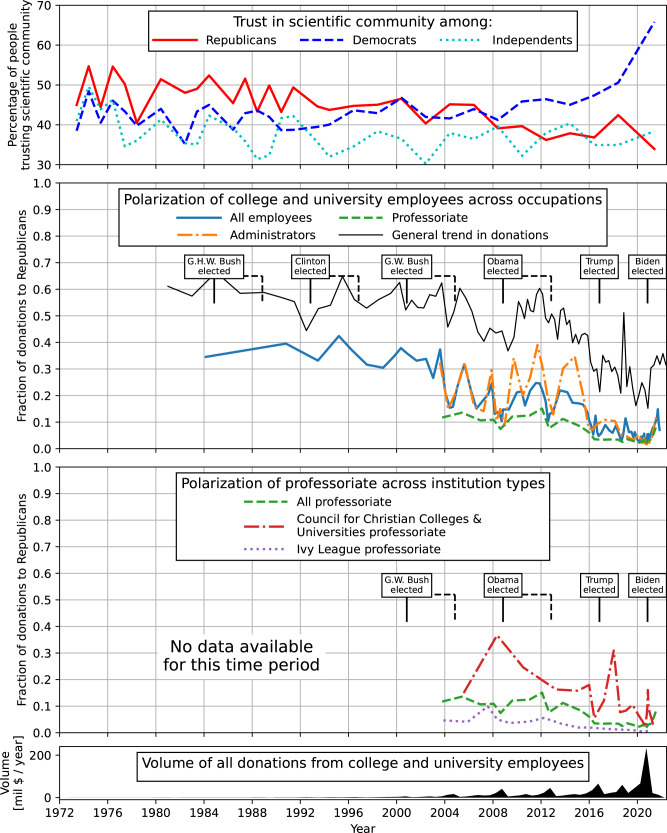
Polarization of college and university employees across occupations. Top panel: fraction of respondents with “a great deal” of confidence in the scientific community among Republicans and Democrats and Independents (neither, no response) in the General Social Survey. The second panel: fraction of donations going to Republican candidates and organizations from all individuals employed by all colleges and universities (solid blue); by the professoriate within those organizations (dashed green); and by the administrators (dot-dashed orange). The general trend in donations to Republican candidates (solid gray) are shown for reference. The third panel: same line for professoriate (dashed green) and further split into professors at institutions affiliated with the Council for Christian Colleges & Universities (dot-dashed red); and by the professoriate at Ivy League institutions (dotted purple). Bottom panel: the total dollar value of donations in million USD per year among all college and university employees. The lines begin when sufficient data are available to evaluate the statistics.

Analysis of the FEC data confirms that American scientists who donate to political candidates favor Democratic candidates and organizations over Republican ones. In fact, they do so dramatically. However, this is a relatively recent phenomenon. From 1984–2000, the proportion of donations to Republicans among all university and college employees was fairly stable, at around 40 percent. Academic employees favored Democrats, but only slightly. (Data are not available to separately analyze scientists vs. other academic employees before 2002.) But, from 2000–2021, donations to Republicans fell drastically, to less than 10 percent (Fig. 1). Starting in 2016, professors gave even less to Republicans than did university employees overall, with only about 5 percent of donations from the professoriate going to Republicans. Ivy League professors gave less still—about 2 percent. The total dollar value of aggregate donations increased dramatically in 2019, when academic donations to Republicans were at a recent historic low. Thus, we can observe that in the past 3 years, academic scientists’ giving has gone almost entirely to Democratic candidates.

**Fig. 2:**
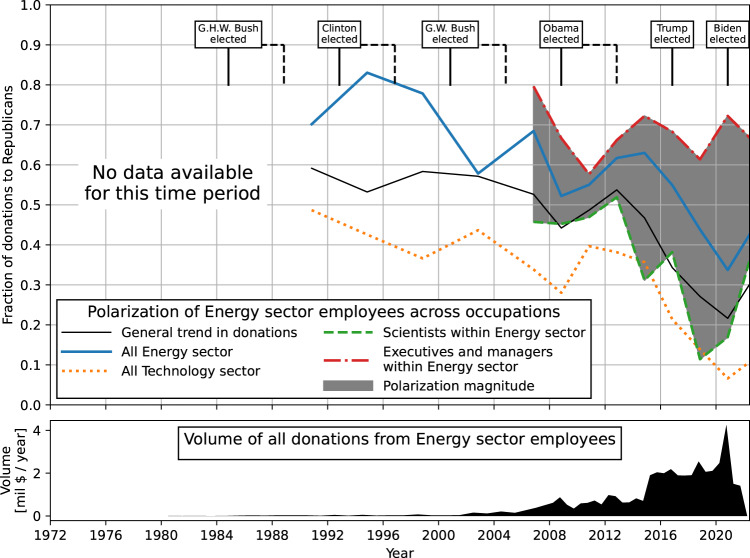
Polarization of Energy sector employees across occupations. Fraction of donations going to Republicans from scientists working in different subject areas: physical sciences (solid green), health sciences (dashed red), social sciences (dot-dashed purple), life sciences (dotted brown).

The data reveal additional striking trends. Professors, including scientists, donate substantially less to Republicans than do other employees of academic institutions, such as administrators. This suggests that administrators, who run their institutions and make many of the policy decisions, are less liberal (or, at least, less estranged from Republicans) than the professors they employ. Second, the trend of decreasing donations to Republicans holds for both sectarian and non-sectarian institutions. While the fraction of total donations to Republicans by researchers at Ivy League institutions has historically been lower than the average for colleges and universities overall, we observe a comparable trend of decreased giving to Republicans from scientists at institutions affiliated with the Council for Christian Colleges and Universities. Third, a decrease in the fraction of donations to the Republican Party can be observed across different disciplines (Fig. 2) (while we observe that the fraction of donations to the Republican Party is slightly higher for professors in the physical sciences than in the health, social and life sciences, to assess variation across scientific disciplines would require improved methods for matching the FEC and SCOPUS databases). These data show that U.S. scientists in academia have in the past 20 years become increasingly estranged from the Republican Party, irrespective of their institution type and discipline.

### Scientists in industry

Slightly less than half (43 percent as of 2019) of American PhD-holding scientists are employed in academia; a similar proportion are employed in the private sector (about 42 percent) (Langin, 2019). Therefore, any analysis of the views of “scientists” that looks only at academics will at best capture half the story.

Here, we focus on the energy sector as a historically conservative-leaning sector that employs large numbers of scientists. We might expect scientists in this sector to be more conservative, and therefore more supportive of the Republican Party, than scientists in academia. While scientists in the energy sector are more supportive of the Republican Party than are their colleagues in academia, the data show a similar pattern of decreasing donations to the Republican Party from energy-sector scientists and engineers.

**Fig. 3:**
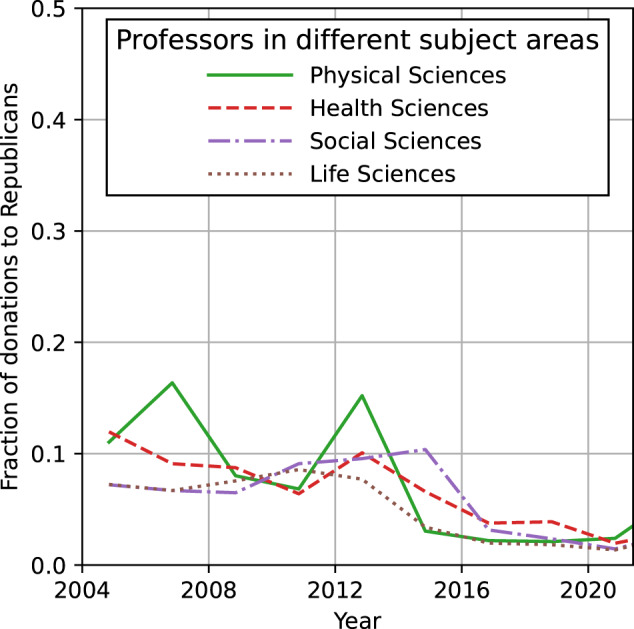
Donations by professors in different subject areas. Fraction of donations going to Republicans from all individuals employed by one of the top 10 energy-sector companies (solid blue), energy-sector scientists and engineers (dashed green), energy-sector executives (dot-dashed red), and the magnitude of polarization between them (shaded region). The donations from all employees in top 10 technology-sector companies are shown for comparison (dotted orange). The general trend in donations to Republican candidates (solid gray). The lower part of the graph indicates the volume of donations in million USD per year among all employees of the top 10 energy-sector companies.

In the energy sector overall, support for Republican candidates decreased from 60 percent of total donations during 1990–2000 to 30 percent in 2020. This decrease is especially marked among energy-sector scientists. Donations from energy-sector scientists to Republicans decreased from 50 percent in 2008–2012 to just 10 percent in 2018–2020. Note that while the fraction of donations to Republicans increases after 2020, the total volume of donations is very low in the post-election period (Fig. 3).

In studying political donations from the energy sector, we see a very large divergence between executives and management, on the one hand, and scientists and engineers, on the other, with executives giving five times more frequently to Republicans during the 2020 election cycle (Fig. 3). Through FEC data we can, in effect, track a cadre of professional scientists who are dissenting from the corporation’s view (Coll, 2013). A comparable divergence (between administrators and the professoriate) prevails in academia, albeit of much lower magnitude. In both industry and academia, working scientists are much less likely to donate money to the Republican Party and Republican candidates than are the people who run their organizations.

### Donations to third parties

**Fig. 4:**
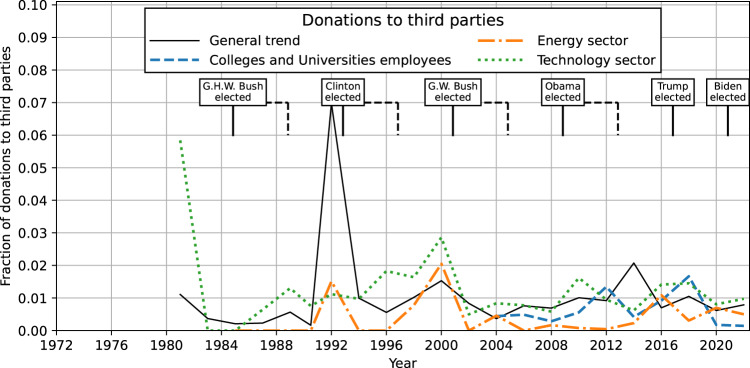
Donations to third parties. Fraction of donations going to third parties from all individuals (solid black/gray), college and university employees (dashed blue), individuals employed by one of the top 10 energy-sector companies (dot-dashed orange), and employees in top 10 technology-sector (dotted green).

Throughout the time period of available data, third-party campaigns and candidates have received only a small fraction of all donations. Given the large share of scientists who identify as political independents, this fact may itself be striking. In Fig. 4, trend lines are shown for various occupational categories used in previous figures. While it is impossible to observe any fine details in trends due to the small number of donations, it is safe to say that none of the trends in third-party giving have shown any major changes comparable to the decline in Republican Party support in recent decades. This stable fraction of around 1% is quite low, but it is comparable to the Republican Party’s support among the professoriate in recent years (Fig. 1).

## Discussion

### Political leanings of scientists and trust in science

Political-donation data confirm that scientists who make donations to political parties or candidates give disproportionately to the Democratic Party. However, this is not a peculiar feature of academic life: scientists in industry, including in the historically conservative energy sector, also donate disproportionately to Democrats.

The fact that scientists in academia and the energy sector predominantly donate to the Democratic Party confirms prevailing impressions and previous survey data that scientists, particularly academic scientists, are more liberal than Americans in general. However, this finding is not proof of a problematic “bias” in academic life. First, a similar pattern holds in industry, yet we rarely hear complaints of liberal bias in industry. Second, prior research shows a strong correlation between political orientation and education level (Geiger, 2016). Sociologists have shown that higher education has a liberalizing effect on social and political views, even while universities also play an important role in the formation of political identity for young conservatives (Gross, 2013; Binder and Wood, 2013; Kidder and Binder, 2020). Science education in particular has been linked to greater political liberalism (Ma-Kellams et al., 2014). Therefore, on average, we would expect scientists in both academia and industry, as people with substantially more education than most Americans, to be substantially more liberal than the general population; the FEC data are consistent with this expectation. Education as a controlling factor would also explain both why scientists in academia and industry are more liberal than their supervisors, and why the polarization magnitude is greater in industry: scientists almost always hold PhDs, university administrators may or may not, and corporate executives in the energy industry rarely do. However, it may also be that the hierarchical position of academic administrators and energy industry executives affects their views on authority, which in turn would affect political preferences.

What education levels do not explain is the dramatic decline in support for Republicans in recent years. We argue that several other factors, beyond education polarization, help to explain the evolving political orientation of scientists, and their recent shift away from the Republican Party—developments that have important implications for public trust in science.

First is the Republican Party’s explicit turn away from science. In recent years, the Republican Party as a whole, and prominent leaders within it, have displayed an antagonistic attitude toward the scientific enterprise and many of its findings, particularly in well-publicized areas of environmental science and public health. This has been true for some time with respect to climate change, with many Republican leaders expressing skepticism about the scientific consensus on climate change and its human causes and some involved in overt attacks on climate scientists such as Michael Mann. In the past 2 years, it has been true with respect to the SARS-CoV2 virus as well, with many Republican leaders challenging scientific findings on the efficacy of masking and vaccination and personal attacks by right-wing institutions and news outlets on Dr. Anthony Fauci. It seems likely that such attacks have alienated many scientists—including those with moderate or conservative views on social, fiscal, or military issues—from the Republican Party (Chris Mooney, 2007).

Second is the Republican Party’s “populist” turn, particularly since the 2016 election of Donald Trump. Conservative attacks on science go beyond complaints about technocracy or excessive regulation. One strategy of Republican populism has been to cast scientists as out-of-touch elites. Conservatives have pushed a portrayal of scientists as politically radical, or as blinkered denizens of the left-wing Ivory Tower, undermining scientific credibility on various issues from the mechanisms driving anthropogenic climate change to public-health guidance against Covid-19. While our analysis focuses on developments in American political life, recent studies by Austrian and Norwegian researchers have also found an association between right-wing populism and negative attitudes toward science (Huber et al., 2021; Krange et al., 2021).

A third factor concerns changes among Democrats. Researchers have charted an increase of trust in science among Democrats since the 1970s. In the same period, and perhaps as a response to Republicans’ attacks on science, Democratic Party elites have frequently incorporated support for science and scientific research into their political messaging. One study has argued that more than half of the increase in the partisan gap in trust in science is due to increased confidence in science among Democrats (Lee, 2021).

Another hypothesis is that the underlying norms of science (specifically communism and universalism) conflict with conservative views. Communism refers to the idea that results of scientific research should be the common property of the scientific community, while universalism refers to the idea that knowledge should transcend racial, class, national, or political barriers. A recent study provides empirical evidence for this hypothesis, showing that endorsement of these values was negatively associated with conservative and libertarian views (Lewandowsky & Oberauer, 2021). Moreover, a substantial literature in political psychology has found that liberals, as compared to conservatives, score higher on measures of tolerance of ambiguity, integrative complexity, and actively open-minded thinking (the last of which involves a commitment to changing one’s mind in response to new evidence) (Azevedo and Jost, 2021; Jost, 2017; Pennycook & Cheyne, 2020; Price et al., 2015). Therefore, certain foundational norms of modern science, as well as certain habits of mind crucial for scientific inquiry, might sit in tension with conservative attitudes. On the other hand, this conflict would not explain the recent character of the observed shift.

The General Social Survey (GSS) provides additional relevant evidence. The GSS has had a question about “confidence in the scientific community,” dating back to the 1970s. In these data, we see that there is both a major ideological shift and a partisan *reversal* of attitudes toward science since the 1970s, and that the substantive changes mostly occurred from 2004 onward (Fig. 1, top panel). As the sociologist Gordon Gauchat has observed, conservatives begin the period with the highest levels of confidence in the scientific community, relative to liberals and moderates, and end with the lowest (Gauchat, 2012). In the 1980s, Republicans were more likely than Democrats to say that they had “a great deal” of confidence in the scientific community. By the 2010s, Democrats were far more likely than Republicans to express a great deal of trust in the scientific community; in 2021, 65 percent of Democrats reported a great deal of confidence in the scientific community, but only 32 percent of Republicans did.

We know of no evidence showing that the makeup of scientists, in terms of their educational levels, changed during this time. In contrast, many studies have tracked how the Republican Party has developed a more oppositional attitude toward science since the Reagan administration (Oreskes & Conway, 2011). Our data show that the partisan reversal in attitudes toward the scientific community roughly overlaps with scientists turning away from the Republican Party. Moreover, as we have shown elsewhere, conservative complaints about “liberal bias” go back into the 1950s, long predating the shift in political donations from scientists revealed by the FEC data (Tyson & Oreskes, 2020). Thus, the evidence does not support the conclusion that Republican voters (or other conservatives) distrust science because scientists are anti-Republican. Rather, it suggests that scientists have turned away from the Republican Party because of its distrust or antagonism to science, particularly in the past 15–20 years.

### Does it matter that scientists support democrats more than republicans?

Is the political orientation of scientists harmful for the validity of scientific results, public trust in science, prospects for the public funding of science, or the perceived legitimacy of science in policymaking? Claims that scientists distort research for the sake of liberal political agendas have not been substantiated by any solid evidence (Larregue, 2018). For instance, one study looking at political slant in psychology research (a field largely dominated by liberal scholars) found that findings of research articles whose abstracts were coded as liberal (i.e., articles whose research conclusions were more consistent with a liberal worldview) were just as likely to be replicable and as statistically robust as the findings of research articles whose abstracts were coded as having a conservative slant (Reinero et al., 2020).

Gallup and Pew surveys confirm the GSS’s finding that Democrats have higher confidence in science than Republicans, and that confidence in science among Republicans has decreased in recent decades (Jones, 2021; Funk et al., 2019). Most Americans, including moderate and liberal Republicans, view scientists as neither conservative nor liberal (Kennedy & Funk, 2015). An increasing share of conservative Republicans, however, perceives scientists as liberal: while in 2009, 28 percent of conservative Republicans saw scientists as liberal, in 2014 that figure had risen to 42 percent (Kennedy & Funk, 2015). Therefore, *perception* of scientists as stalwarts of the opposing party may well have contributed to Republicans’ decreased trust in science, especially in a context of increasing party polarization.

Republicans’ decreased trust in science is further driven by opposition to the policy implications of certain scientific findings (e.g., anthropogenic drivers of climate change)—especially demands for greater government regulation or new forms of taxation to address the problem. Such resistance might explain why 73 percent of Democrats believe that scientists should take an active role in policy debates, compared to 43 percent of Republicans (Kennedy and Funk, 2019).

The distance that conservatives have maintained from academic science may also confer certain political advantages. By establishing intellectual networks, especially think tanks, that operate outside the confines of the academy, conservative intellectuals have avoided retreating into scholasticism and have succeeded in pushing public debate to the right (Medvetz, 2014; Gross et al., 2011).

### What can scientists do?

If the trends captured in our study continue, the increased clustering of scientists away from Republicans might further undermine the perceived legitimacy of scientists qua policy advisors in the view of conservative voters, and further decrease conservative Republicans’ trust in science. These trends might lead some people to consider that perhaps conservatives are right when they call for greater ideological diversity in academic life. However, that solution, if it can be called that, neglects the role of Republican anti-scientific positions.

What might make sense, however, is to focus attention on scientists whose views are more in line with Americans in general. The data examined here pertain only to scientists who gave money subject to FEC disclosure rules. Scientists who give money to political parties likely feel strongly about political issues, and it may be that these scientists are also more likely to contribute to public conversations on contested issues. If so, it would reinforce the common impression that scientists, like other academics, are all extremely liberal.

However, as discussed above, available evidence shows that most academics are actually politically moderate or only slightly left of center. There are likely many scientists of moderate or conservative political persuasion who support causes other than Republican candidates and the Republican Party, perhaps in part because they are troubled by prevailing Republican positions on evolution, climate, or Covid-19. Many of these scientists may be sitting on the sidelines, as it were, of public debate. Studies of the “trusted messenger effect” suggest that we are all more likely to trust a message if it comes from someone who we believe shares our values (Siegrist et al., 2000; Kahan, 2010). Therefore, we suggest that it may help public understanding of science, and perhaps improve trust in science among Republicans, if the scientists not captured in this study did more to engage in public conversation and debate in order to create a conversational climate of value pluralism (Cologn, 2021). For example, a recent study found that Republicans’ understanding of the existence, causes, and harms of climate change increased when exposed to professional videos featuring Republican spokespeople sharing climate messages that resonate with conservative values (Goldberg et al., 2021). Therefore, greater engagement by Republican scientists might help foster trust in science among Republicans and push back against anti-scientific messaging from the Republican Party’s elites.

## Data Availability

All data used in this study are publicly available on the following websites: Federal Election Commission (FEC.gov, 2022): https://www.fec.gov/data/browse-data/?tab=bulk-data. The General Social Survey (Smith, 2015): https://gss.norc.org/. SCOPUS API by Elsevier (Elsevier, 2022): https://dev.elsevier.com/
